# Bombali Ebolavirus in *Mops condylurus* Bats (Molossidae), Mozambique

**DOI:** 10.3201/eid2812.220853

**Published:** 2022-12

**Authors:** Camille Lebarbenchon, Steven M. Goodman, Axel O.G. Hoarau, Gildas Le Minter, Andréa Dos Santos, M. Corrie Schoeman, Christophe Léculier, Hervé Raoul, Eduardo S. Gudo, Patrick Mavingui

**Affiliations:** Processus Infectieux en Milieu Insulaire Tropical, University of Reunion, Inserm, CNRS, IRD, Saint-Denis, Reunion Island, France (C. Lebarbenchon, A.O.G. Hoarau, G. Le Minter, P. Mavingui);; Field Museum of Natural History, Chicago, USA S.M. Goodman);; Association Vahatra, Antananarivo, Madagascar (S.M. Goodman);; Eduardo Mondlane University, Maputo, Mozambique (A. Dos Santos);; University of Kwa-Zulu Natal, Kwa-Zulu Natal, South Africa (M.C. Schoeman);; Jean Mérieux Inserm P4 Laboratory, Lyon, France (C. Léculier, H. Raoul);; National Institute of Health, Maputo, Mozambique (E.S. Gudo)

**Keywords:** Bombali ebolavirus, Mops condylurus, Mozambique, viruses

## Abstract

We detected Bombali ebolavirus RNA in 3 free-tailed bats (*Mops condylurus*, Molossidae) in Mozambique. Sequencing of the large protein gene revealed 98% identity with viruses previously detected in Sierra Leone, Kenya, and Guinea. Our findings further support the suspected role of *Mops condylurus* bats in maintaining Bombali ebolavirus.

Six viruses of the genus *Ebolavirus* have been documented to date (Zaire, Sudan, Bundibugyo, Taï Forest, Reston, and Bombali), and some have caused outbreaks in Africa, resulting in high human fatality rates. Bombali virus (BOMV) was first identified in free-tailed bats of the family Molossidae, specifically the species *Mops condylurus* and *Chaerephon pumilus*, in 2016 in the Bombali District in Sierra Leone (*1*). This virus was later detected in *M. condylurus* bats in Kenya (*2*,*3*) in 2018 and in Guinea (*4*) in 2019 (Figure, panel A). Human infections have not been documented, including in patients with febrile illness symptoms in areas where BOMV has been found in bats (*2*).

**Figure:**
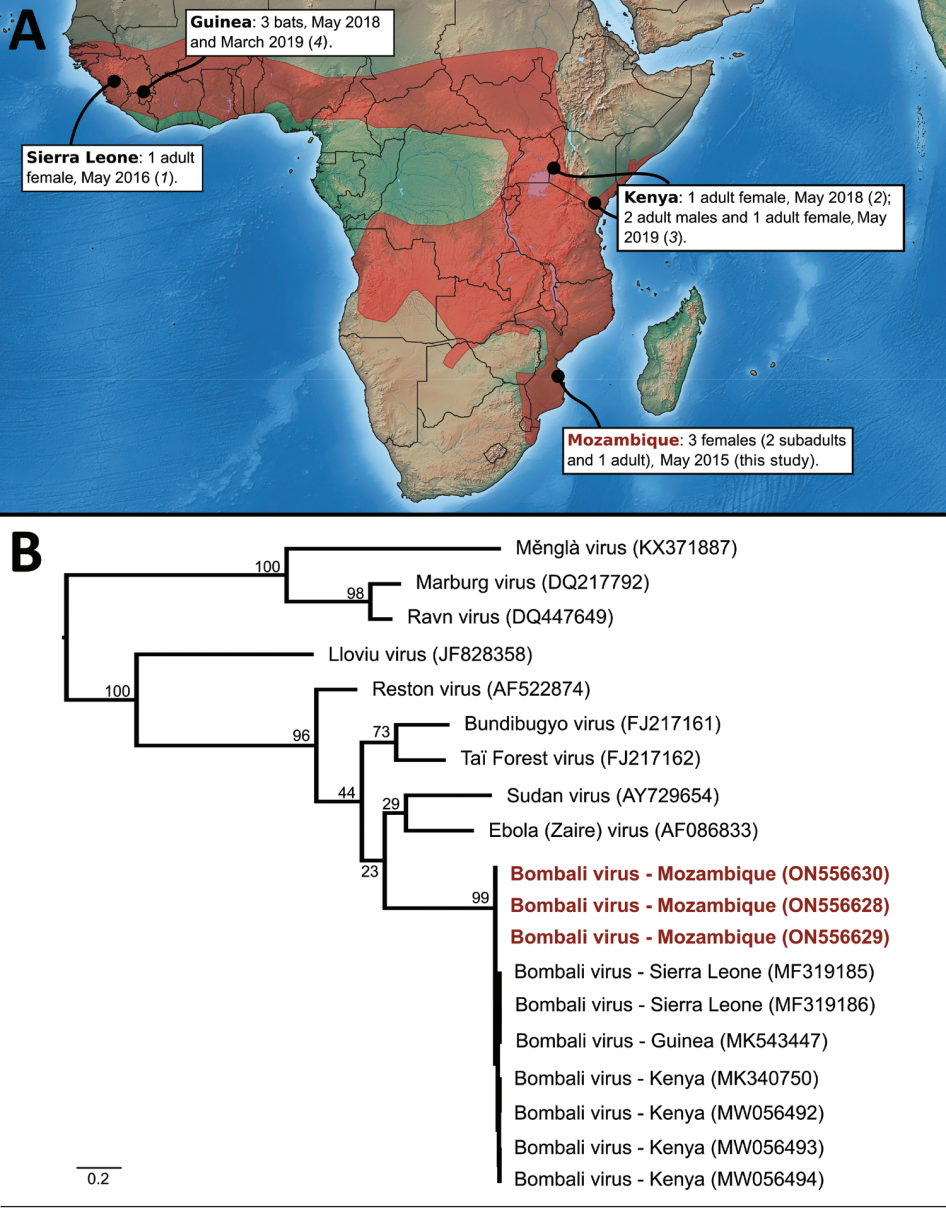
Bombali virus detection in Angolan free-tailed bats (*Mops condylurus*). A) Geographic range highlighted in red. Information regarding the sex of positive *M. condylurus* bats in Guinea is not available (*4*). The map was generated with data available from Natural Earth (https://www.naturalearthdata.com) and the International Union for Conservation of Nature Red List Web site (https://www.iucnredlist.org). B) Maximum-likelihood tree based on partial nucleotide sequences (587 bp) of the large protein gene of selected filoviruses. Red indicates sequences generated in this study. The phylogenetic analysis was conducted with the transversion plus gamma evolutionary model (α = 0.32) and 1,000 bootstraps (Appendix). All but 1 of the Bombali virus were detected in *Mops condylurus* bats, with the exception of MF319186, which collected from a *Chaerephon pumilus* bat (*1*).

We detected BOMV RNA in 3 *M*. *condylurus* bats (all female) captured in Mozambique in the southeastern portion of this species’ geographic range (Figure, panel A). In May 2015, we obtained samples from 54 *M. condylurus* bats residing in buildings in the Inhassoro District of southeastern Mozambique and from 211 other bats (representing 10 species), mostly from caves (Appendix). We screened all samples for viruses belonging to the families *Astroviridae* (*5*), *Coronaviridae* (*6*), and *Paramyxoviridae* (*7*). We performed RNA extraction with the QIAamp Viral RNA Mini Kit (QIAGEN, https://www.qiagen.com) and reverse transcription with the ProtoScript II Reverse Transcriptase and Random Primer 6 (New England BioLabs, https://www.neb.com). We screened complementary DNA with 3 assays targeting the large (L) protein gene of Filoviriridae (Appendix) and submitted PCR products of the expected size for direct Sanger sequencing (GenoScreen, https://www.genoscreen.fr). We did not attempt virus isolation in this study. We processed samples in a Biosafety Level 3 laboratory at the University of Reunion (Saint-Denis, Reunion Island, France) and transferred original samples to the Biosafety Level 4 laboratory at Inserm Jean Mérieux (Lyon, France).

To date, BOMV is the only ebolavirus that has been recurrently detected by PCR, across multiple years (2015–2019), and in bat populations located >5,000 km apart (*1*–*4*). Our study provides support for BOMV in the southern range of where *M*. *condylurus* bats are known to reside (Figure, panel A). Partial sequencing of the L protein gene revealed that the BOMV sequences detected in bats from Mozambique were closely related to those sequences reported in bats from Sierra Leone, Kenya, and Guinea (Figure, panel B). Although our findings are based on short sequences (587 bp), this finding could suggest a strong association between BOMV and *M. condylurus* bats across their geographic range.

BOMV epidemiology in *M*. *condylurus* bats is unknown. Seasonal variation of environmental conditions and population structure are important drivers for the transmission dynamics of infectious agents in natural systems (*8*). For instance, pulses of Marburg virus, paramyxovirus, and coronavirus shedding have been shown to coincide with a seasonal increase of juveniles in bat populations (*9*,*10*). Although our study was based on a limited sampling, we detected BOMV only in female bats (χ^2^ = 4.6; df = 1; p<0.05; no. tested females/males: 26/28) and did not find differences between adults and subadults (χ^2^ = 0.5; df = 1; p = 0.46; no. tested adults/subadults: 29/25). Previous reports likewise reported BOMV more frequently in female bats (Figure, panel A) (*1*–*3*⁠). All prior studies reported BOMV-positive bats during the month of May (Figure, panel A) (*1*–*4*⁠). Whether these observations reflect a biologic phenomenon remains to be tested. Across their geographic range, female *M*. *condylurus* bats usually have 2 birthing periods that occur between September and early May, and some variation in virus shedding can be anticipated with each reproductive cycle, as documented for other bat–virus systems. Longitudinal studies are needed to investigate biologic and ecologic factors involved in the transmission dynamics of BOMV in *M. condylurus* bats but also to fully assess virus spillover risk to other hosts, including humans.

In Mozambique, neither BOMV nor other species of ebolavirus have been detected in humans, highlighting that our findings should not be considered evidence of a major threat to local communities, but should be instead considered a catalyst for further investigation and surveillance. Additional studies should focus on other Molossidae bats, because BOMV was initially reported in another member of the family, *Chaerephon pumilus* (*1*), and these bats commonly roost in synanthropic settings and therefore generate opportunities for spillover. Indeed, most BOMV-positive bats were captured in day-roost sites in buildings occupied by humans or livestock (*1*,*3*,*4*). Assessing livestock exposure to BOMV also would be prudent, given their key role as intermediate hosts in the emergence of zoonotic viruses.

The discovery of ebolavirus in wild animals raises questions regarding virus spillover and epidemic potential. In addition to the identification of molecular factors involved in the ability of the virus to replicate in human cells, risk assessment should include environmental factors across the local, social, and habitat landscape. Employing a One Health approach (i.e., collaborative, multisectoral, and transdisciplinary) might prevent future outbreaks, promote sustainable development of human communities, and offer protection for bats that play a key functional role in ecosystems.

Appendix Additional information on bombali ebolavirus in *Mops condylurus* bats (Molossidae), Mozambique.
