# Supplementary material for: Bombali Ebolavirus in Mops condylurus Bats (Molossidae), Mozambique
Source: Emerg Infect Dis. 2022 Dec;28(12):2583–5. doi: 10.3201/eid2812.220853 (PMC9707587; doi:10.3201/eid2812.220853)
Supplement: Appendix — Additional information on bombali ebolavirus in Mops condylurus bats (Molossidae), Mozambique. [file 22-0853-Techapp-s1.pdf]

Article DOI: https://doi.org/10.3201/eid2812.220853

Bombali Ebolavirus in *Mops condylurus* bats (Molossidae), Mozambique

Appendix

Bat sampling

Bats were sampled in February and in May 2015, as described previously (*1*). Briefly, bats were captured using mist nets and harp traps. Bat species, sex and age were determined based on different morphological characters. For each individual bat, one rectal and one buccal swab were collected. The two swabs were then placed in a single tube containing 1.5 ml of Brain Heart Infusion media (Conda, Madrid, Spain) supplemented with penicillin G (1000 units/ml), streptomycin (1 mg/ml), kanamycin (0.5 mg/ml), gentamicin (0.25 mg/ml), and amphotericin B (0.025 mg/ml). Samples were immediately frozen in liquid nitrogen. Number of sampled bats per species is provided in the Appendix Table.

Bat sampling was approved by the Reunion Island Animal Care and Use Committee and authorized by the French Ministry of Education and Research (Reference numbers 03584.01 and APAFIS#2638-2015110616208322v1). In Mozambique, research permits were issued by the Museum of Natural History (Ref. 01/MHN/E.27/2015) and the Ministry of Health (N°S/N/SDI/0233/15).

Molecular analysis

Samples were vortexed and centrifuged at 1500 g for 15 min. Virus inactivation was done with AVL buffer and nucleic acid extraction performed with the QIAamp Viral RNA Mini Kit (QIAGEN, Valencia, CA, USA). Reverse-transcription was performed on 10 µL of RNA, with the ProtoScript II Reverse Transcriptase and Random Primer 6 (New England BioLabs, Ipswich, MA, USA), under the following thermal conditions: 70°C for 5 min, 25°C for 10 min, 42°C for 50 min and 65°C for 20 min (*2*). cDNA were screened with three assays targeting the large protein gene of *Filoviriridae* (*3*), Zaire ebolavirus (*4*), and BOMV (*3*). PCRs were performed

with the GoTaq G2 Hot Start Green Master Mix (Promega, Madison, WI, USA) in an Applied Biosystems 2720 Thermal Cycler (Thermo Fisher Scientific, Waltham, MA, USA). Electrophoresis was done on 1.5% agarose gels stained with 2% GelRed (Biotium, Hayward, CA, USA). Real-time PCR were performed with a CFX96 Touch Real-Time PCR Detection System (Bio-Rad, Hercules, CA, USA).

Phylogenetic analysis

Nucleotide sequences were aligned with representative sequences of *Filoviridae* based on the International Committee on Taxonomy of Viruses classification. A 587 bp alignment was obtained with Geneious Prime 2022.0.1 (Biomatters Ltd.), and a maximum-likelihood analysis was conducted using PhyML 3.1 (*5*) with a sequence evolutionary model selected by Model Generator (*6*) and 1000 bootstraps. Sequences generated in this study are available in GenBank under the ON556628 to ON556630 accession numbers.

References

1. Hoarau AOG, Goodman SM, Al Halabi D, Ramasindrazana B, Lagadec E, Le Minter G, et al. Investigation of astrovirus, coronavirus and paramyxovirus co-infections in bats in the western Indian Ocean. Virol J. 2021;18:205. PubMed https://doi.org/10.1186/s12985-021-01673-2

2. Lebarbenchon C, Ramasindrazana B, Joffrin L, Bos S, Lagadec E, Le Minter G, et al. Astroviruses in bats, Madagascar. Emerg Microbes Infect. 2017;6:1. PubMed https://doi.org/10.1038/emi.2017.47

3. Goldstein T, Anthony SJ, Gbakima A, Bird BH, Bangura J, Tremeau-Bravard A, et al. The discovery of Bombali virus adds further support for bats as hosts of ebolaviruses. Nat Microbiol. 2018;3:1084–9. PubMed https://doi.org/10.1038/s41564-018-0227-2

4. Jääskeläinen AJ, Moilanen K, Aaltonen K, Putkuri N, Sironen T, Kallio-Kokko H, et al. Development and evaluation of a real-time EBOV-L-RT-qPCR for detection of Zaire ebolavirus. J Clin Virol. 2015;67:56–8. PubMed https://doi.org/10.1016/j.jcv.2015.04.003

5. Guindon S, Dufayard J-F, Lefort V, Anisimova M, Hordijk W, Gascuel O. New algorithms and methods to estimate maximum-likelihood phylogenies: assessing the performance of PhyML 3.0. Syst Biol. 2010;59:307–21. PubMed https://doi.org/10.1093/sysbio/syq010

6. Keane TM, Creevey CJ, Pentony MM, Naughton TJ, Mclnerney JO. Assessment of methods for amino acid matrix selection and their use on empirical data shows that ad hoc assumptions for choice of matrix are not justified. BMC Evol Biol. 2006;6:29. PubMed https://doi.org/10.1186/1471-2148-6-29

Appendix Table. Mozambican bat species tested for the presence of Bombali Ebolavirus.

Family	Species	Tested	Positive
Hipposideridae	*Hipposideros caffer*	59	0
Miniopteridae	*Miniopterus mossambicus*	21	0
Molossidae	*Mops condylurus*	54	3
Nycteridae	*Nycteris thebaica*	14	0
Rhinolophidae	*Rhinolophus lobatus*	9	0
	Rhinolophus mossambicus	20	0
	Rhinolophus rhodesiae	31	0
	Rhinolophus sp.	2	0
Rhinonycteridae	*Triaenops afer*	51	0
Vespertilionidae	*Neoromicia nana*	2	0
	Scotophilus viridis	2	0
